# Identifying the Subfamilies of Voltage-Gated Potassium Channels Using Feature Selection Technique

**DOI:** 10.3390/ijms150712940

**Published:** 2014-07-22

**Authors:** Wei-Xin Liu, En-Ze Deng, Wei Chen, Hao Lin

**Affiliations:** 1 Key Laboratory for Neuro-Information of Ministry of Education, Center of Bioinformatics, School of Life Science and Technology, University of Electronic Science and Technology of China, Chengdu 610054, China; E-Mails: lweixin316@gmail.com (W.-X.L.); enzeas@gmail.com (E.-Z.D.); 2 Department of Physics, School of Sciences, and Center for Genomics and Computational Biology, Hebei United University, Tangshan 063000, China; E-Mail: greatchen@heuu.edu.cn

**Keywords:** voltage-gated potassium channel, subfamily, optimized tripeptide composition, support vector machine, feature selection

## Abstract

Voltage-gated K^+^ channel (VKC) plays important roles in biology procession, especially in nervous system. Different subfamilies of VKCs have different biological functions. Thus, knowing VKCs’ subfamilies has become a meaningful job because it can guide the direction for the disease diagnosis and drug design. However, the traditional wet-experimental methods were costly and time-consuming. It is highly desirable to develop an effective and powerful computational tool for identifying different subfamilies of VKCs. In this study, a predictor, called iVKC-OTC, has been developed by incorporating the optimized tripeptide composition (OTC) generated by feature selection technique into the general form of pseudo-amino acid composition to identify six subfamilies of VKCs. One of the remarkable advantages of introducing the optimized tripeptide composition is being able to avoid the notorious dimension disaster or over fitting problems in statistical predictions. It was observed on a benchmark dataset, by using a jackknife test, that the overall accuracy achieved by iVKC-OTC reaches to 96.77% in identifying the six subfamilies of VKCs, indicating that the new predictor is promising or at least may become a complementary tool to the existing methods in this area. It has not escaped our notice that the optimized tripeptide composition can also be used to investigate other protein classification problems.

## 1. Introduction

Ion channels located in the surface of cell membrane can maintain the balance of cell microenvironment by selectively penetrating ions and organic molecules in and out of cells. The K^+^ channel has been found in all living organisms [1]. The voltage-gated K^+^ channel (VKC), which is the largest family of K^+^ channels, specifically controls the movement of K^+^ under the stimulation of voltage changes in the cell's membrane potential. During action potentials, they play crucial roles in returning the depolarized cell to a resting state [2]. They are also key components in generation and propagation of electrical impulses in nervous system. The mutations in VKC genes can lead to severe diseases, such as long QT syndrome and epilepsy [3]. Thus, VKCs have become valuable targets for disease diagnosis and drug design.

**Figure 1:**
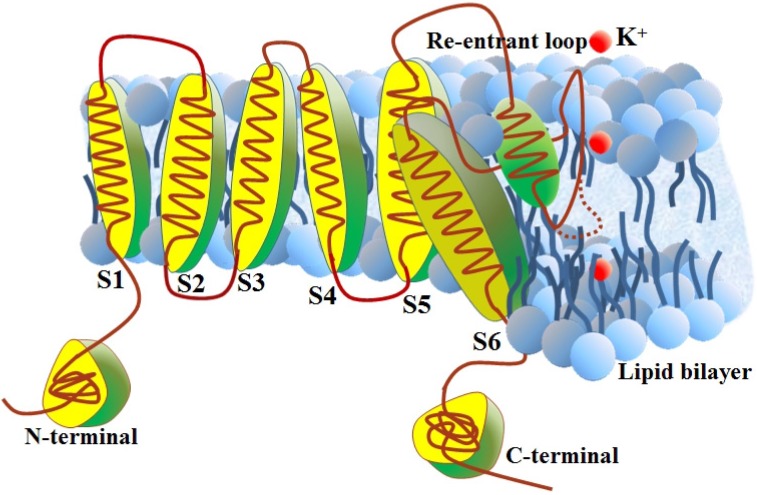
Schematic representation of potassium (K^+^) channel subunit. The S1, S2, S3, S4, S5, S6 are six transmembrane helices.

VKCs have four subunits. Each subunit comprises six transmembrane helices. A re-entrant loop forms the ion-selective channel, highly variable *C*- and *N*-terminal domains (Figure 1). According to the *N*- and *C*-terminal domains, VKCs can be grouped into different subfamilies. The proteins in these subfamilies are functionally divergent. Different subfamilies of VKC proteins have different sensitivity to the membrane potential and response to changes in potential [2]. Therefore, recognition of subfamily type of a new VKC is benefit to understand its biological functions. However, the traditional biochemical methods were costly and time-consuming. Thus, it is necessary to develop effective computational methods to identify subfamilies of VKCs.

In the past decade, some scholars have focused on the identification of VKCs families. Liu *et al.* [4] proposed a dipeptide-based method to predict five subfamilies of VKCs. Subsequently, Chen and Lin [5] developed an SVM-based model to predict six subfamilies of VKCs by using the Correlation-based Feature Subset Selection algorithm (CFSS) to select the optimal features. All these methods could yield quite encouraging results, and each of them did play a role in stimulating the development of this area. However, further work is needed due to the following reasons. (i) The predicted successful rate is still far from satisfaction; (ii) No web-server was provided to most of these methods, and, hence, their usage is quite limited, especially for the majority of experimental scientists.

The present study was initiated in an attempt to improve the prediction of VKC subfamilies from the above two aspects. According to a comprehensive review [6], to establish a really useful statistical predictor for VKC subfamily prediction, an objective benchmark dataset was constructed. Subsequently, a feature selection technique was used to obtain the optimal tripeptides. The support vector machine was used to operate the prediction. The jackknife cross-validation test was utilized to estimate the accuracy of the predictor. Finally, we established a user-friendly web-server for the predictor.

## 2. Results and Discussion

### 2.1. Benchmark Dataset

The raw dataset of VKCs were extracted from the updated Voltage-gated K^+^ Channel Database (VKCDB) [2] and filtered by VKCPred [5]. The following steps were used to construct a reliable benchmark dataset. At first, if the primary structure (amino acid sequence) of a VKC contains ambiguous residues, such as “B”, “X”, and “Z”, the VKC will be removed; Secondly, if the sequence is fragment of other proteins, it will be excluded because its information is redundant and fragmentary; Thirdly, to objectively evaluate the proposed predictor, the CD-HIT software [7] was used to remove highly similar sequences by setting the cutoff of sequence identity to 60%. As a result, we obtained the benchmark dataset S as formulated by:

S = S_1_ ∪ S_2_ ∪ S_3_ ∪ S_4_ ∪ S_5_ ∪ S_6_ (1)

where the subset S_1_ contains 82 Kv1 subfamily proteins, S_2_ contains 16 Kv2 subfamily proteins, S_3_ contains 37 Kv3 subfamily proteins, S_4_ contains 32 Kv4 subfamily proteins, S_5_ contains 10 Kv6 subfamily proteins and S_6_ contains 40 Kv7 subfamily proteins (Table 1) and where U represents the symbol for union in the set theory. For readers’ convenience, the 217 VKCs can be freely downloaded from our webserver.

**Table 1:** Breakdown of the 217 voltage-gated K^+^ channels (VKCs) in the benchmark dataset S according to their six subfamilies.

Dataset	Channel Subfamilies	Number of VKC Samples
S_1_	Kv1	82
S_2_	Kv2	16
S_3_	Kv3	37
S_4_	Kv4	32
S_5_	Kv6	10
S_6_	Kv7	40
S	Overall	217

### 2.2. The Tripeptide Composition

To develop a sequence-based predictor for the prediction of the subfamilies of VKCs, one of the keys is to formulate its sequence with an effective mathematical expression that can truly reflect the intrinsic correlation with the types to be predicted. The most straightforward method to formulate the sample of a VKC protein P with *L* residues is to use its entire amino acid sequence, as can be formulated by:

P = R_1_R_2_R_3_R_4_…R*_L_* (2)

where R_1_ represents the 1st residue of the protein P, R_2_ represents the 2nd residue of the protein P, and so forth. According to a recent review [8], the general form of PseAAC for a protein P is formulated by:

P = [Ψ_1_ Ψ_2_ … Ψ*_u_* … Ψ_Ω_]^T^ (3)

where the subscript Ω is an integer and its value, as well as the components Ψ*_u_* (*u* = 1, 2, …, Ω), will depend on how to extract the desired information from the amino acid sequence P (*cf.* Equation (3)).

Tripeptide is a useful and minimal biological recognition signal which can be used for studying molecular modulators of biological function [9] and predicting plausible structures for oligopeptides as well as de novo protein design [8]. Thus, we extract tripeptide composition from the benchmark dataset S to define the components in Equation (3) for the VKC samples concerned in this study. Then a VKC sequence can be formulated by:

P = [*f_1_*, *f_2_*, … *f_i_*, … *f_8000_*]^T^ (4)

where symbol T denotes the transposition of vector and the *f_i_* is the frequency of the *i*-th (*i* = 1, 2, …, 8000) tripeptide in the VKC and expressed as:

*f_i_* = *n_i_*/(*L* − 2) (5)

where *n_i_* and *L* denote the occurrence number of the *i*-th tripeptide and the length of the VKC sequence, respectively.

### 2.3. Feature Selection

If all 8000 tripeptides are used for prediction, the predictive result isn’t usually satisfactory, such as low generalization ability of prediction model and poor prediction results because irrelevant features and noise is included. On the other hand, it is time-consuming to analyze an 8000 dimensional vector for large amounts of proteins. Using feature selection techniques to optimize feature set can not only gain deeper insight into the intrinsic properties of VKCs, but also improve understandability, scalability, possibility, and accuracy of the proposed models. Moreover, it can also economize the time for model construction and prediction.

Although many dimensionality reduction techniques such as principal component analysis (PCA) [10,11], diffusion Maps [12] and minimal-redundancy-maximal-relevance (mRMR) [13,14] have been proposed to perform feature selection, none of them concerned the statistical significance of the features. According to this, we proposed the binomial distribution to investigate the statistical significance of each tripeptide and the optimal the feature set.

Each of the 8000 tripeptides occurring in one subfamily may be a stochastic event, thus, we must calculate the confidence level (*CL*) of each tripeptide occurring in different VKC subfamilies. For a stochastic event, two possible cases that are occurrence and non-occurrence will happen when one observes the *i*-th tripeptide occurring in the *k*-th VKC subfamily. Each outcome has a fixed probability when benchmark dataset has been fixed. This probability is called prior probability and defined as:

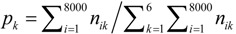
(6)
where 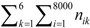 denotes the total occurrence number of all tripeptides in the benchmark dataset. 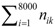 is the occurrence number of all tripeptides in the *k*-th VKC subfamily. The *n_ik_* represents the number of the *i*-th tripeptides occurring in the *k*-th VKC subfamily. Correspondingly, the probability of the non-occurrence in the *k*-th VKC subfamily is defined as *q_k_* = 1 − *p_k_*.

Let 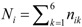 represents the total occurrence number of the *i*-th tripeptide in benchmark dataset. That is to say, under the condition of the prior probability *p_k_*, one performs trial or observation with *N_i_* times. We may calculate the posterior probability *P_ik_* of the *i*-th tripeptide occurring *n_ik_* or more times in the *k*-th VKC subfamily as following:

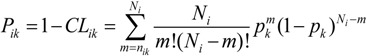
(7)
where *CL_ik_* is the *CL* of the *i*-th tripeptide in the *k*-th VKC subfamily. Based on small probability event principle, if *P_ik_* is a small value, it means the tripeptide *i* appearing in VKC subfamily *k* is not random.

There are six VKC subfamilies in the current study, namely *k* = 1, 2, 3, 4, 5, 6. Hence, for an arbitrary tripeptide *i*, it has six *CLs* corresponding to six VKC subfamilies. Then, we may define the probability of tripeptide *i* in benchmark dataset as:

*CL_i_* = max {*CL_i Kv1_*, *CL_i Kv2_*, *CL_i Kv3_*, *CL_i Kv4_*, *CL_i Kv5_*, *CL_i Kv6_*, *CL_i Kv7_*} (i = 1,2,…,8000) (8)

It should be noted that the larger the *CL_i_* is, the more likely this feature has a better discriminative capability. Therefore, we ranked the tripeptides according to their *CL_i_*. Based on the ranked tripeptides, we used the Incremental Feature Selection (IFS) strategy to find an optimal subset of features that gives the highest overall accuracy. During the IFS procedure, the feature subset started with one feature with the largest *CL*. A new feature subset was composed when one feature with the second largest *CL* had been added. By adding features one by one from larger to smaller rank, this process repeated 8000 times until all the features were evaluated. Thus, the 8000 feature sets thus formed would be composed of 8000 ranked features. The *τ*-th feature set can be formulated as:

*S_τ_* = {*f_1_*, *f_2_*, … *f_i_*, … *f_τ_*} (1 ≤ *τ* ≤ 8000) (9)

where *f_i_* has been defined by Equation (5). For each of the feature sets, the cross-validation test was used to investigate the accuracy by using proposed predictive algorithm. Through the method referred above, we got an IFS curve in Descartes Curvilinear Coordinate System, which used τ as *X* axis, *CL* as *Y* axis and overall accuracy as *Z* axis. The optimal feature set is expressed as:

*S_Θ_* = {*f_1_*, *f_2_*, … *f_i_*, … *f_Θ_*} (10)

with which the IFS curve reaches its peak. In other words, in the 3D Cartesian coordinate system, when *X* = Θ, the value of overall accuracy is the maximum. Thus, we used the Θ features to build the final predictor.

### 2.4. Support Vector Machine

Support vector machine has been widely applied in bioinformatics [15,16,17,18,19,20]. The basic idea of applying SVM to pattern classification is to map samples with low dimensional feature space into a high dimensional space, and then seek an optimal separating hyperplane with the maximal margin in this space by using the decision function:

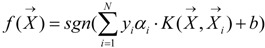
(11)
where 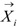 is the *i*-th training vector. The *y_i_* represents the type of the *i*-th training vector. *α_i_* is coefficient which can be solved by quadratic programming. The *b* is the intercept parameter. 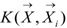 is a kernel function which defines an inner product in a high dimensional feature space. Because of its effectiveness and speed in nonlinear classification process, the radial basis kernel function (RBF) 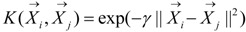 was used to in this work.

The traditional SVM was designed for two-class problems. For handling a multi-class problem, “one-versus-one (OVO)” and “one-versus-rest (OVR)” are often applied to extend the traditional SVM. The present study adopted OVO strategy for multi-class prediction. The software toolbox used to implement SVM is LibSVM [21]. A grid search method was used to optimize the regularization parameter *c* and kernel parameter *γ* by using cross-validation test. The search spaces for *c* and *γ* are (2^15^, 2^−5^) and (2^−5^, 2^−15^) with steps being 2^−1^ and 2, respectively.

### 2.5. Prediction Assessment

The predictive capability and reliable of the method is estimated by the four parameters: the sensitivity (*Sn*), specificity (*Sp*), Matthew’s correlation coefficient (*MCC*) and overall accuracy (*OA*), which were employed to measure the performance of the method and can be defined as follows:

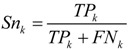
(12)

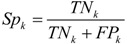
(13)

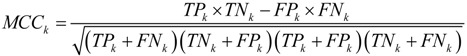
(14)

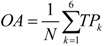
(15)
where *k* is the *k*-th VKC subfamily, *N* is the total sequence number of benchmark dataset. *TP_k_*, *TN_k_*, *FP_k_* and *FN_k_* represent true positive, true negative, false positive and false negative of the *k*-th VKC subfamily, respectively.

## 3. Experimental

In statistical prediction, the following four cross-validation test methods were often used to build a predictor for its effectiveness in practical application: self-consistency test, independent dataset test, *n*-fold cross-validation and jackknife cross-validation. Among them, the jackknife test method makes best use of the data, involves no random sub-sampling and achieves unique results [6,22]. It has been widely and increasingly adopted in bioinformatics [5,12,13,14,23,24,25]. Therefore, the jackknife cross-validation was used in all procession of feature selection and parameter optimization of SVM.

Based on Equations (4)–(5), we may define the 8000 tripeptide composition as the original feature set. Generally, the larger the feature set is, the more information the representation bears. However, the tripeptides with low *CL* (or large posterior probability) maybe randomly appear in six VKC subfamilies. Including these tripeptides into feature set will add redundant information or reduce the cluster-tolerant capacity so as to lower down the cross-validation accuracy. For example, 8000 tripeptides can only produce the overall accuracy of 92.17% for predicting different VKC subfamilies. In contrast, the tripeptides with larger *CL* (or small posterior probability) give more reliable information for classification. The occurrence of these tripeptides prefers to different VKC subfamilies. However, if the number of tripeptide in feature set is very small, they are still not the optimized features for prediction because they cannot reflect real characteristics of VKCs and afford enough information, which deduces the poor predictive accuracy. For instance, by selecting 29 tripeptides with *CL*~100% (*p* value = 10^−^^7^), we can only achieve 81.10%.

**Figure 2:**
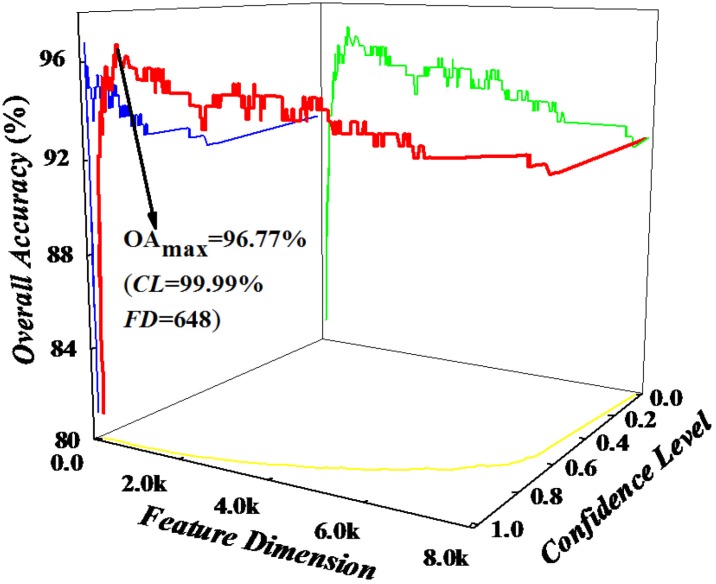
The IFS curve (red) in a 3D Cartesian coordinate system for predicting six subfamilies of VKCs. The blue, green and yellow lines are the projections of the IFS curve on the Overall accuracy/Confidence level plane, the Overall accuracy/Feature dimension plane, the Feature dimension/Confidence level plane, respectively.

Therefore, it is a key step to obtain the best feature set which can product the maximum overall accuracy. According to the equation from Equation (6) to (9), we calculated the cross-validated accuracy of all 8000 feature sets using SVM and plotted a three-dimensional curve for *CL*, feature dimension and OA in Figure 2. As we can see from Figure 2, the overall accuracy reaches its maximum of 96.77% when the *CL* is selected as 99.99%. The optimized feature set contains 648 tripeptides. Results in Table 2 show that the average *Sn* and average *Sp* are 93.92% and 99.20%, respectively, indicating that the proposed method is indeed very powerful in identifying proteins which belongs to different subfamilies of VKCs.

Recently, the optimized dipeptide composition (DPC) and amino acid composition (AAC) selected by Correlation-based Feature Subset Selection (CFSS) algorithm were used to predict six VKC subfamilies by Chen and Lin [5]. In jackknife cross-validation, the overall accuracies of 93.09%, 85.71% and 82.03% were obtained by SVM, Naïve Bayes and Random Forest, respectively. The comparative results in Table 2 demonstrate that the method proposed in this paper is superior to the published methods [5].

**Table 2:** Comparison with other published methods.

Family	This Paper			SVM [ 5]			Naïve Bayes [ 5]			Random Forest [ 5]		
	*Sn* (%)	*Sp* (%)	*MCC*	*Sn* (%)	*Sp* (%)	*MCC*	*Sn* (%)	*Sp* (%)	*MCC*	*Sn* (%)	*Sp* (%)	*MCC*
Kv1	100.00	96.30	0.95	93.90	93.98	0.86	93.90	83.85	0.76	97.56	78.51	0.76
Kv2	93.75	100.00	0.96	87.50	98.95	0.86	81.25	100.00	0.89	75.00	98.78	0.82
Kv3	97.30	98.89	0.95	89.19	97.69	0.87	81.08	95.12	0.75	59.45	97.44	0.67
Kv4	100.00	100.00	1.00	93.75	100.00	0.96	87.50	100.00	0.92	65.38	98.73	0.75
Kv6	80.00	100.00	0.89	100.00	100.00	1.00	40.00	100.00	0.62	80.00	98.82	0.87
Kv7	92.50	100.00	0.95	95.00	99.39	0.95	85.00	98.70	0.87	85.00	99.29	0.89
Average *Sn* (%)	93.92			93.22			78.12			77.07		
Average *Sp* (%)	99.20			98.34			96.28			95.26		
*OA* (%)	96.77			93.09			85.71			82.03		

For verifying the advantage of optimized tripeptide composition, it is necessary to investigate the performance of other parameters. Hence, we estimated the accuracies of traditional pseudo amino acid composition (PseAAC) [6], optimal tripeptides combined with PseAAC and optimal tripeptides combined with dipeptides on six subfamilies of voltage-gated ion channels. Results were recorded in Table 3. It is obviously that the optimized tripeptide composition is superior to other parameters. It should be noted that the two mixture features can only achieve the overall accuracies of 96.31% and 95.39% which are lower than that (96.77%) of our optimal tripeptides, suggesting that information redundancy or noise were included in mixture feature sets.

For testifying the capability of the proposed feature selection technique, a powerful feature selection technique, namely SVM-RFE [26,27], was introduced to optimize the tripeptides. Subsequently, the IFS strategy was used to find an optimal subset of features that gives the highest overall accuracy. The maximum accuracy was recorded in Table 3. Comparison demonstrated that our feature selection technique is more powerful.

**Table 3:** Comparison with different methods on training set.

Method	Sn (%)						OA (%)
	Kv1	Kv2	Kv3	Kv4	Kv6	Kv7	
Optimal tripeptides (Our method)	100.00	93.75	97.30	100.00	80.00	92.50	96.77
Optimal tripeptides (SVM-RFE)	100.00	81.25	91.67	96.88	80.00	87.55	93.09
Traditional PseAAC	82.93	81.25	72.97	78.13	80.00	87.50	81.11
Optimal tripeptides (Our method) + PseAAC	100.00	87.50	97.30	100.00	80.00	92.50	96.31
Optimal tripeptides (Our method) + Dipeptides	100.00	81.25	94.59	100.00	80.00	92.50	95.39

## 4. Conclusions

In this work, we developed a promising feature selection technique to optimize feature set and applied these selected features to identify six VKC subfamilies. An overall accuracy of 96.77% was achieved, demonstrating that the proposed model is a powerful tool for the study of VKC subfamilies prediction. For the convenience of experimental scientists, a free web server iVKC-OTC was built to implement the prediction. A friendly guide was given to describe the way to use the iVKC-OTC web server. We believe that the predictor will be helpful for wet lab scientists who focus on VKC research. We hope the predictor will pave the way for the future research of VKC.

## 5. Web-Server and User Guide

Establishing a user-friendly web-server will improve the efficiency and avoid repeating a complicated mathematics and program for studying VKC. The predictor established via aforementioned procedures is called iVKC-OTC, where “i” stands for “identify”, “VKC” for “Voltage-gated K^+^ channel” and “OTC” for “optimized tripeptide composition”.

For the convenience of the vast majority of experimental scientists, we provided a guide on how to use the web-server to get the desired results.

**Figure 3:**
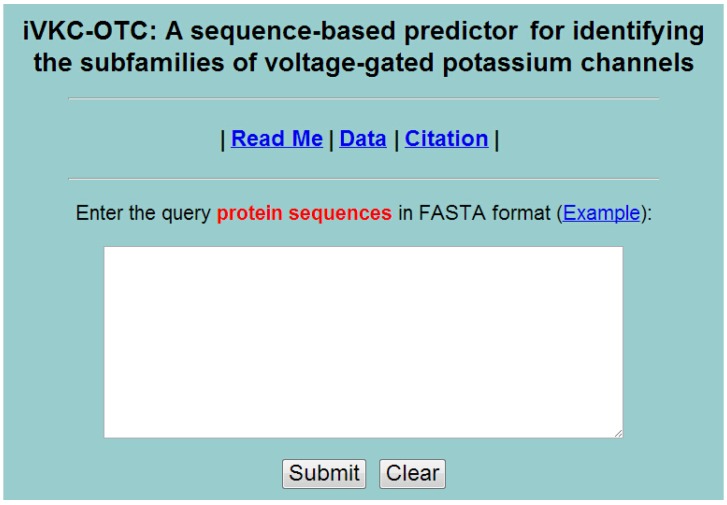
A semi-screenshot for the top page of the iVKC-OTC.

Step 1. Open the web server and you will see the top page of iVKC-OTC [28] on your computer screen, as shown in Figure 3 Click on the Read Me button to see a brief introduction about the predictor and the caveat when using it.

Step 2. Either type or copy/paste the query peptide sequences into the input box at the center of Figure 3 The input sequence should be in the FASTA format. A sequence in FASTA format consists of a single initial line beginning with a greater-than symbol (“>”) in the first column, followed by lines of sequence data. The words right after the “>” symbol in the single initial line are optional and only used for the purpose of identification and description. The sequence ends if another line starting with a “>” appears; this indicates the start of another sequence. Example sequences in FASTA format can be seen by clicking on the Example button right above the input box.

Step 3. Click on the Submit button to see the predicted result. After clicking the Submit button, you will see the following shown on the screen of your computer: the outcome for the 1st query example is “Kv1 subfamily protein”; the outcome for the 2nd query sample is “Kv2 subfamily protein”; the outcome for the 3rd query sample is “Kv3 subfamily protein”; the outcome for the 4th query sample is “Kv4 subfamily protein”; the outcome for the 5th query sample is “Kv6 subfamily protein” and the outcome for the 6th query sample is “Kv7 subfamily protein”. All these results are fully consistent with the experimental observations. It takes about few seconds for the above computation before the predicted result appears on your computer screen; the more number of query sequences and longer of each sequence, the more time it is usually needed.

Step 4. Click on the Data button to download the benchmark datasets used to train and test the iVKC-OTC predictor.

Step 5. Click on the Citation button to find the relevant papers that document the detailed development and algorithm of iVKC-OTC.

Caveats. Each of the input query sequences cannot any illegal character: such as “B”, “X”, “U”, “Z”.
